# Mathematical Modeling and Control of Infectious Diseases

**DOI:** 10.1155/2017/7149154

**Published:** 2017-12-07

**Authors:** Gul Zaman, Il H. Jung, Delfim F. M. Torres, Anwar Zeb

**Affiliations:** ^1^Department of Mathematics, University of Malakand, Chakdara, Pakistan; ^2^Department of Mathematics, Pusan National University, San 30, Busan 609-735, Republic of Korea; ^3^Department of Mathematics and CIDMA, University of Aveiro, Aveiro, Portugal; ^4^Department of Mathematics, COMSATS Institute of Information Technology, Abbottabad, Pakistan

Mathematical modeling has become a valuable tool for the analysis of dynamics of infectious disease and for the support of control strategies development in recent years. This work highlights the conceptual ideas and mathematical tools needed for infectious diseases modeling. The main convergence of this was on the dynamics of infectious diseases, the analysis of transmission patterns in various populations, and methods to assess the effectiveness of control strategies such as HIV, childhood infections, influenza, and vector borne infections. It was concerned with qualitative behaviors of infectious disease model. The qualitative behavior of model includes positivity, uniqueness, local stability, global stability, bifurcation analysis, control of diseases, and existence of solutions. This study provided a platform for the discussion of the major research challenges and achievements on qualitative behaviors of infectious diseases and their control. Due to the availability of a lot of applications of this study, many authors contributed.

G. R. Phaijoo and D. B. Gurung demonstrated that dengue is spreading in new areas due to people movement. They considered a multipatch model to assess the impact of temperature and human movement on the transmission dynamics of dengue disease. Dynamics of vector and host populations are investigated with different human movement rates and different temperature levels.

N. Pipatsart et al. discussed adaptive random network models to describe human behavioral change during epidemics and performed stochastic simulations of SIR epidemic models on adaptive random networks.

C. Burgess et al. derived risk-based immunization by deployment to polio-endemic regions, which is sufficient to prevent transmission among both deployed and nondeployed US military populations.

T. Tilahun et al. analyzed a compartmental nonlinear deterministic mathematical model for the typhoid fever outbreak and optimal control strategies in a community with varying population.

L. Worden et al. extended an approximation technique of the long-term behavior of a supercritical stochastic epidemic model, using the WKB approximation and a Hamiltonian phase space, to the subcritical case.

S. Anis et al. discussed some of the genetic properties on the basis of algebra.

Finally, A. Miao et al. proposed a stochastic SIR model with vertical transmission and vaccination. They showed that large noise can lead to the extinction of infectious diseases, which is conducive to epidemic diseases control.

*Gul Zaman* *Il H. Jung* *Delfim F. M. Torres* *Anwar Zeb*

